# Food Poisoning and *Staphylococcus aureus* Enterotoxins

**DOI:** 10.3390/toxins2071751

**Published:** 2010-07-05

**Authors:** María Ángeles Argudín, María Carmen Mendoza, María Rosario Rodicio

**Affiliations:** Department of Functional Biology (Section of Microbiology) and University Institute of Biotechnology of Asturias (IUBA), University of Oviedo, Oviedo, Spain; Email: argudinmaria@gmail.com (M.A.A.); cmendoza@uniovi.es (M.C.M)

**Keywords:** *Staphylococcus aureus*, food poisoning, staphylococcal enterotoxins, emetic activity, superantigens, gene location

## Abstract

*Staphylococcus aureus* produces a wide variety of toxins including staphylococcal enterotoxins (SEs; SEA to SEE, SEG to SEI, SER to SET) with demonstrated emetic activity, and staphylococcal-like (SE*l*) proteins, which are not emetic in a primate model (SE*l*L and SE*l*Q) or have yet to be tested (SE*l*J, SE*l*K, SE*l*M to SE*l*P, SE*l*U, SE*l*U2 and SE*l*V). SEs and SE*l*s have been traditionally subdivided into classical (SEA to SEE) and new (SEG to SE*l*U2) types. All possess superantigenic activity and are encoded by accessory genetic elements, including plasmids, prophages, pathogenicity islands, *v*Sa genomic islands, or by genes located next to the staphylococcal cassette chromosome (SCC) implicated in methicillin resistance. SEs are a major cause of food poisoning, which typically occurs after ingestion of different foods, particularly processed meat and dairy products, contaminated with *S. aureus* by improper handling and subsequent storage at elevated temperatures. Symptoms are of rapid onset and include nausea and violent vomiting, with or without diarrhea. The illness is usually self-limiting and only occasionally it is severe enough to warrant hospitalization. SEA is the most common cause of staphylococcal food poisoning worldwide, but the involvement of other classical SEs has been also demonstrated. Of the new SE/SE*l*s, only SEH have clearly been associated with food poisoning. However, genes encoding novel SEs as well as SE*l*s with untested emetic activity are widely represented in *S. aureus*, and their role in pathogenesis may be underestimated.

## 1. Staphylococcal Food Poisoning

Staphylococcal food poisoning (SFP) is an intoxication that results from the consumption of foods containing sufficient amounts of one (or more) preformed enterotoxin [1,2]. Symptoms of SFP have a rapid onset (2–8 h), and include nausea, violent vomiting, abdominal cramping, with or without diarrhea [3,4,5]. The disease is usually self-limiting and typically resolves within 24–48 h after onset. Occasionally it can be severe enough to warrant hospitalization, particularly when infants, elderly or debilitated people are concerned [4].

Food handlers carrying enterotoxin-producing *S. aureus* in their noses or on their hands are regarded as the main source of food contamination, via manual contact or through respiratory secretions. In fact, *S. aureus* is a common commensal of the skin and mucosal membranes of humans, with estimates of 20–30% for persistent and 60% for intermittent colonization [6]. Because *S. aureus* does not compete well with indigenous microbiota in raw foods, contamination is mainly associated with improper handling of cooked or processed foods, followed by storage under conditions which allow growth of *S. aureus* and production of the enterotoxin(s). However, *S. aureus* is also present in food animals, and dairy cattle, sheep and goats, particularly if affected by subclinical mastitis, are likely contaminants of milk [7]. Air, dust, and food contact surfaces can also serve as vehicles in the transfer of *S. aureus* to foods.

Foods that have been frequently incriminated in staphylococcal intoxication include meat and meat products, poultry and egg products, milk and dairy products, salads, bakery products, particularly cream-filled pastries and cakes, and sandwich fillings [8,9]. Salted food products, such as ham, have also been implicated [10], according to the capacity of *S. aureus* to grow at relatively low water activity (a_w_ = 0.86; [11]).

SFP is a common disease whose real incidence is probably underestimated for a number of reasons, which include misdiagnosis, unreported minor outbreaks, improper sample collection and improper laboratory examination. The control of this disease is of social and economic importance. In fact, it represents a considerable burden in terms of loss of working days and productivity, hospital expenses, and economical losses in food industries, catering companies and restaurants [2,3,12,13,14,15].

## 2. *Staphylococcus aureus* Enterotoxins

The *S. aureus* enterotoxins (SEs) are potent gastrointestinal exotoxins synthesized by *S. aureus* throughout the logarithmic phase of growth or during the transition from the exponential to the stationary phase [16,17,18,19,20]. They are active in high nanogram to low microgram quantities [21], and are resistant to conditions (heat treatment, low pH) that easily destroy the bacteria that produce them, and to proteolytic enzymes, hence retaining their activity in the digestive tract after ingestion [22,23,24].

**Table 1:** General properties of SEs and SE*l*s and genomic location of the encoding genes. See text for references. nd, not determined; ^a^ Emetic activity demonstrated in rabbits (SE*l*L; [43]) or in the small insectivore *Suncus murinus* (SE*l*P; [39]) but not in a primate model; ^b^ Hypothetical location in a prophage [48].

**Toxin**	**Molecular Mass (kDa)**	**Emetic Activity**	**Crystal Structure Solved**	**Gene**	**Accessory genetic element**
SEA	27.1	yes	yes	*sea*	ΦSa3ms, ΦSa3mw, Φ252B, ΦNM3, ΦMu50a
SEB	28.4	yes	yes	*seb*	pZA10, SaPI3
SEC	27.5–27.6	yes	yes	*sec*	SaPIn1, SaPIm1, SaPImw2, SaPIbov1
SED	26.9	yes	yes	*sed*	pIB485-like
SEE	26.4	yes	no	*see*	ΦSa ^b^
SEG	27.0	yes	yes	*seg*	*egc*1 (*v*Saβ I); *egc*2 (*v*Saβ III); *egc*3; *egc*4
SEH	25.1	yes	yes	*seh*	MGEmw2/mssa476 *seh*/∆*seo*
SEI	24.9	weak	yes	*sei*	*egc*1 (*v*Saβ I); *egc*2 (*v*Saβ III) ); *egc*3
SE*l*J	28.5	nd	no	*selj*	pIB485-like; pF5
SE*l*K	26.0	nd	yes	*selk*	ΦSa3ms, ΦSa3mw, SaPI1, SaPI3, SaPIbov1, SaPI5
SE*l*L	26.0	no ^a^	no	*sell*	SaPIn1, SaPIm1, SaPImw2, SaPIbov1
SE*l*M	24.8	nd	no	*selm*	*egc*1 (*v*Saβ I); *egc*2 (*v*Saβ III)
SE*l*N	26.1	nd	no	*seln*	*egc*1 (*v*Saβ I); *egc*2 (*v*Saβ III); *egc*3; *egc*4
SE*l*O	26.7	nd	no	*selo*	*egc*1 (*v*Saβ I); *egc*2 (*v*Saβ III); *egc*3; *egc*4; MGEmw2/mssa476 *seh*/∆*seo*
SE*l*P	27.0	nd ^a^	no	*selp*	ΦN315, ΦMu3A
SE*l*Q	25.0	no	no	*selq*	ΦSa3ms, ΦSa3mw, SaPI1, SaPI3, SaPI5
SER	27.0	yes	no	*ser*	pIB485-like; pF5
SES	26.2	yes	no	*ses*	pF5
SET	22.6	weak	no	*set*	pF5
SE*l*U	27.1	nd	no	*selu*	*egc*2 (*v*Saβ III); *egc*3
SE*l*U2 (SEW)	nd	nd	no	*selu2*	*egc*4
SE*l*V	nd	nd	no	*selv*	*egc*4

### 2.1. Nomenclature

SEs belong to the broad family of pyrogenic toxin superantigens (SAgs; [3]). SAgs bypass conventional antigen recognition by interaction with major histocompatibility complex (MHC) class II molecules on the surface of antigen presenting cells, and with T-cell receptors (TCR) on specific T-cell subsets. Interaction typically occurs to the variable region of the TCR β chain (Vβ) but binding to the TCR Vα domain has been reported [21,25,29]. This leads to activation of a large number of T-cells followed by proliferation and massive release of chemokines and proinflammatory cytokines that may led to potentially lethal toxic shock syndrome [3]. However, staphylococcal enterotoxins have been proposed to be named according to their emetic activities [30]. Only SAgs that induce vomiting after oral administration in a primate model will be designated as SEs. Related toxins that lack emetic activity or have not been tested for it should be designated as staphylococcal enterotoxin-like (SE*l*s) SAgs. Also, newly discovered toxins with more than 90% amino acid sequence identity with existing SEs or SE*l*s should be designated as a numbered subtype. However, despite this consensus nomenclature some subtypes are still just called variants.

At the time of this review, the repertoire of *S. aureus* SEs/SE*l*s comprised 22 members, excluding molecular variants: (i) the classical SEA, SEB, SEC (with the SEC1, SEC2 and SEC3, SEC ovine and SEC bovine variants), SED and SEE, which were discovered in studies of *S. aureus* strains involved in SFP outbreaks, and classified in distinct serological types [31,32,33,34,35]; and (ii) the new types of SEs (SEG, SEH, SEI, SER, SES, SET) and SE*l*s (SE*l*J, SE*l*K, SE*l*L, SE*l*M, SE*l*N, SE*l*O, SE*l*P, SE*l*Q, SE*l*U, SE*l*U2, and SE*l*V) [28,36,37,38,39,40,41,42,43,44,45]. TSST-1, the toxic shock staphylococcal toxin, initially designated as SEF, lacks emetic activity [46,47].

### 2.2. Structure

SEs and SE*l*s constitute a family of structurally related exoproteins that range in size from ~22 to 28 kDa (Table 1). Based on amino acid sequence comparisons, they have been distributed into four or five groups (Table 2), depending on the inclusion or not of SEH within group 1 [21,29,40,49]. The recently described SET is most related to a putative exotoxin from an *S. aureus* isolate involved in bovine mastitis, and to streptococcal pyrogenic toxin type K (SpeK) [40]. TSST-1, which is functionally a superantigen with no emetic activity, is more distant to SEs and SE*l*s than to SSLs (staphylococcal superantigen-like proteins) [50]. The SSLs, first identified by screening staphylococcal genomes using two conserved amino acid motifs placed in the N-terminal and C-terminal domains of SAgs, are not mitogenic to T cells and do not bind MHC class II, although they display a wide array of activities targeting key elements of the innate and specific immunity, such as neutrophils, complement factor C5, and IgA [51,52,53,54,55,56].

**Table 2:** Grouping of SEs and SE*l*s based on amino acid sequence comparisons. Modified from Larkin *et al.* [21]. Enterotoxins encoded by the *egc* cluster are shown in bold. SEH (in parenthesis) has been placed within Group 1 or Group 5, depending on the author [29,49].

Group	SEs and SE*l*s
Group 1	SEA, SED, SEE, (SEH), SE*l*J, SE*l*N, SE*l*O, SE*l*P, SES
Group 2	SEB, SEC, SEG, SER, SE*l*U, SE*l*U2
Group 3	SEI, SE*l*K, SE*l*L, SE*l*M, SE*l*Q, SE*l*V
Group 4	SET
(Group 5)	(SEH)

The three-dimensional structures of TSST-1 [57,58] and several SEs and SE*l*s [59,60,61,62,63,64,65,66,67,68,69] have been solved by crystallography (Table 1). The structures are remarkably conserved, although they interact differently with MHC class II molecules, and show different TCR specificity [70]. They are compact ellipsoidal proteins with two unequal domains separated by a shallow grove. The larger C-terminal domain is a β-grasp fold consisting of four- to five-strand β-sheet that packs against a highly conserved α-helix [71]. The smaller N-terminal domain consists of a mixed β-barrel with Greek-key topology, similar to the OB (oligosaccharide/oligonucleotide binding)-fold [72] also found in many other bacterial toxins (SSLs, streptococcal superantigens, nucleases and toxins of the AB_5_ family, including cholera and pertussis toxins, and verotoxin) [29,50]. The two domains are stabilized by close packing and by a section of the N-terminus that extends over the top of the C-terminal domain. The N-terminal extension contributes substantially to the TCR-binding site, located in the cleft between the two protein domains, while the MHC class II binding site is in the OB-fold [29,50]. The top of the N-terminal domain usually contains a highly flexible disulfide loop, which has been implicated with emetic activity (see below).

### 2.3. Mode of Action

Important efforts have been made to identify specific amino acids and domains within SEs which may be important for emesis, but results are still limited and controversial. Like TSST-1, SE*l*L, and SE*l*Q are nonemetic, while SEI displays weak emetic activity [38,41,42]. These toxins lack the disulfide loop characteristically found at the top of the *N*-terminal domain of other SEs. Nonetheless, the loop itself does not appear to be an absolute requirement for emesis, although it may stabilize a crucial conformation important for this activity [73]. Carboxymethylation of histidines on SEA or SEB generates proteins devoid of enterotoxicity, which still retain superantigenicity [75,76]. Analysis of the effects of carboxymethylation of each of the SEA histidines revealed that His61 is important for emesis, but not for T-cell proliferation [77]. Conversely, Leu48Gly and Phe44Ser mutant forms of SEA and SEB, respectively, do not bind MHC class II molecules or cause T-cell activation, but still provoke vomiting [78], hence separating emesis and superantigenicity as different functions of the proteins. Despite this, a high correlation exists between the two activities since, in most cases, genetic mutations resulting in a loss of superantigen activity also results in loss of emetic activity [78].

In contrast to the case of many other bacterial enterotoxins, specific cells and receptors in the digestive system have not been unequivocally linked to oral intoxication by a SE. It has been suggested that SEs stimulate the vagus nerve in the abdominal viscera, which transmits the signal to the vomiting center in the brain [79]. Supporting this idea, receptors on vagal afferent neurons are essential for SEA-triggered emesis [80], and capsaicin, a small molecular weight compound from chilli peppers that depletes peptidergic sensory nerve fibers, also diminishes SE effects in mammals [21]. In addition, SEs are able to penetrate the gut lining and activate local and systemic immune responses [81]. Release of inflammatory mediators (including histamine, leukotrienes, and neuroenteric peptide substance P) causes vomiting [82,83,84,85] and the emetic response can be eliminated by H2- and calcium channel-blockers, which also block the release of histamine [86]. Local immune system activation could also be responsible for the gastrointestinal damage associated with SE ingestion [87,88]. Inflammatory changes are observed in several regions of the gastrointestinal tract, but the most severe lesions appear in the stomach and the upper part of the small intestine [89]. The diarrhea sometimes associated with SEs intoxication may be due to the inhibition of water and electrolyte reabsorption in the small intestine [90,91]. In an attempt to link the two distinct activities of SEs, *i.e.*, superantigenicity and enterotoxicity, it has been postulated that enterotoxin activity could facilitate transcitosis, enabling the toxin to enter the bloodstream and circulate through the body, thus allowing the interaction with antigen presenting- and T-cells that leads to superantigen activity [3,92]. In this way, circulation of SEs following ingestion of SEs as well as their spread from a *S. aureus* infection site, could have more profound effects upon the host *versus* if the toxin remains localized [21].

### 2.4. Enterotoxin Gene Location

All *se* and *sel* genes are located on accessory genetic elements, including plasmids, prophages, *S. aureus* pathogenicity islands (SaPIs), genomic island *v*Sa, or next to the staphylococcal cassette chromosome (SCC) elements (Table 1). Most of these are mobile genetic elements, and their spread among *S.* *aureus* isolates can modify their ability to cause disease and contribute to the evolution of this important pathogen.

#### 2.4.1. Plasmids

**Figure 1:**
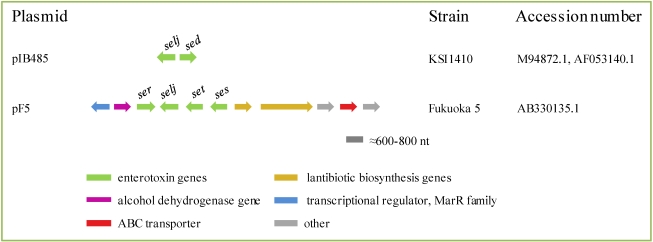
Enterotoxin and enterotoxin-like genes in plasmids pIB485 and pF5 based on sequencing data deposited under the accession numbers indicated to the right of the figure. Note thatpIB485 also contains *blaZ* and *cad* resistance genes [94] and probably *ser* [40,95].

Plasmids have been long recognized as efficient vehicles for the spread of resistance and virulence determinants through horizontal gene transfer. In *S. aureus*, two kinds of plasmids carrying *se*/*sel* genes have been characterized (Table 1; Figure 1). Both contain *sej* and *ser* associated with either *sed* (pIB485-like) or with *ses* and *set* (pF5) [40,45,93].

The first plasmid described to carry an enterotoxin gene was pIB485, a 27.6 kilobase (kb) plasmid, in which first *sed* and latter *selj* were identified [45,94]. Enterotoxin SER was discovered by [93] in *S. aureus* strains associated with a food poisoning outbreak that occurred in Fukuoka City, Japan, in 1997, and the *ser* gene was shown to be located on a family of closely related plasmids, termed pF5 and pF5-like. These plasmids have similar restriction profiles and carry *selj* along with *ser*. More recently, two novel SE genes (*ses* and *set*) have also been detected on the Fukuoka plasmids [40,93]. Interestingly, the *ser* gene, together with *sed* and *selj*, has also been found in pIB485-like plasmids from laboratory strains, food poisoning outbreak isolates and healthy human isolates in Japan [93] and pIB485-like plasmids, varying in size and/or restriction profile were present in *S. aureus* isolates recovered in Spain from human nasal carriers and manually handled foods [95]. Two of them, named pUO-Sa-SED1 (~33 kb) and pUO-Sa-SED2 (~36 kb), carried *sed*, *selj* and *ser*, and have restriction patterns identical or similar to that of pIB485, while pUO-Sa-SED3 (53.5 kb; containing *sed*, *selj* and *ser*-like) has a different profile. A BLAST search (http://www.ncbi.nlm.nih.gov) of the *sed*, *selj*, *ser*, *ses* and *set* genes revealed additional pIB485-like and pF5-like plasmids obtained from human clinical isolates, whose sequences have been deposited in databases. At present, the evolutionary relationship between the two types of plasmids is unknown.

#### 2.4.2. Prophages

**Figure 2:**
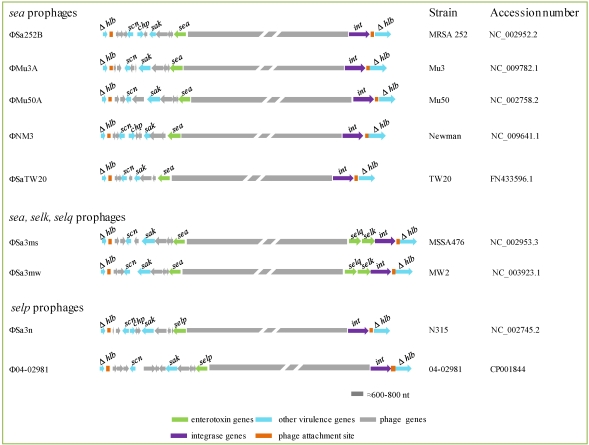
Enterotoxin genes carried by prophages based on sequencing data deposited under the accession numbers indicated to the right of the figure.

Like most published *S. aureus* phages, those carrying *se* genes (*sea*, *selk*, *selp* and *selq*) belong to the *Siphoviridae* family. The temperate, tailed bacteriophages within this family have been classified according to three features [96]: (i) the lysogeny module, particularly the integrase that dictates the insertion site of the phage in the bacterial chromosome; (ii) the serogroup, based on differences in capsid, tail, and tail appendix proteins; and (iii) the holin gene of the lysis module. The *Siphoviridae* prophages carrying *se* genes belong to integrase group Sa3, serogroups Fa and Fb, and holin groups 255a and 255b. Three *se*/*sel* genes (*sea*, *selk* and *selq*) are present together in ФSa3ms and ФSa3mw, while a single *se*/*sel* gene (*sea* or *selp*) is carried by other prophages (Table 1; Figure 2). 

Apart from enterotoxins, virulence factors involved in evasion of the innate immunity are also encoded on these phages. These include the chemotaxis inhibitory protein (CHIP, product of the *chp* gene) that binds to host chemokine receptors, particularly the C5a receptor and the formylated peptide receptor, preventing neutrophil chemotaxis and activation [97]; the staphylococcal complement inhibitor (SCIN, encoded by the *scn* gene) that interferes with all pathways of complement activation by blocking C3 convertases [98]; the staphylokinase (product of the *sak* gene) that leads to degradation of two major opsonins (IgG and C3b) through activation of surface-bound plasminogen into plasmin, and also inhibits the bactericidal effect of α-defensins [99,100]. The region encoding these virulence factors is known as the "innate inmune evasion cluster" [101] and is located at one or both ends of the phages. Integration of these phages into the *S. aureus* chromosome occurs by a site-specific recombination event between the *attP* site in the phage genome and the *attB* site located within the β-hemolysin gene in the bacterial chromosome [102]. While integration negatively converts β-hemolysin expression, it supplies other virulence genes.

#### 2.4.3. *Staphylococcus aureus* Pathogenicity Islands

The SaPIs are mobile pathogenicity islands, which are widely distributed in *S. aureus* and have also been found in other species of *Staphylococcus*. SaPIs have a highly conserved overall organization, parallel to that of typical temperate bateriophages. Each one occupies a specific chromosomal site (att*_S_*), and always appears in the same orientation. From its integration site, the island can be induced to excise and replicate by one or more specific staphylococcal helper phages [103,104]. Following replication the SaPI DNA is efficiently encapsidated into infectious small-headed phage-like particles resulting in extremely high transfer frequencies.

**Figure 3:**
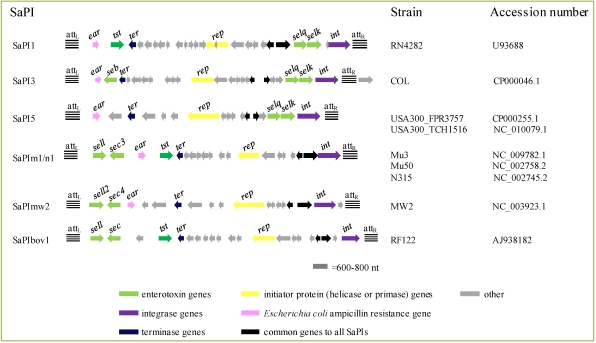
*Staphylococcus aureus* pathogenicity islands (SaPIs) carrying enterotoxin or enterotoxin-like genes. Modified from Novick and Subedi [105] and based on the accession numbers indicated to the right of the figure.

SaPIs are very common in *S. aureus* (Table 1). They range in size from 15–17 kb, with the exceptions of SaPIbov2 (27 kb) and a highly degenerated SaPI (3.14 kb) present in some sequenced genomes. The complete nucleotide sequence is known for 20 SaPIs, and some of them carry genes encoding TSST-1 and/or one or more SEs (Figure 3). For instance, *tst* is found together with *selk* and *selq* in SaPI1, with *sec3* and *sell* in SaPIm1 and SaPIn1, and with *sell* and *sec* in SaPIbov1; *seb*, *selq* and *selk* have been reported in SaPI3; *selk* and *selq* in SaPI5; and *sec4* and *sell2* in SaPImw2 [105]. Induction of a SaPI is likely to originate an increase in the copy number of the toxin genes, and therefore to an increase in toxin production, as described for lysogenic phages [106].

#### 2.4.4. *v*Sa G*enomic Islands*

The term *v*Sa refers to non-phage and non-SCC genomic islands that are exclusively present in *S. aureus*, often (but not always) encode virulence determinants, are inserted at specific loci in the chromosome and are associated with either intact or remnant DNA recombinases [107,108]. Two major *v*Sa genomic islands, namely *v*Saα and *v*Saβ, each of about 20–30 kb, are present in all *S. aureus* genomes sequenced so far, but absent in other *Staphylococcus* species, including *S. epidermidis*. Though *v*Saα and *v*Saβ could have been acquired by horizontal gene transfer, actually there is not evidence that they can move. Each of these islands carries two copies of the genes encoding the recognition (*hsdS*) and methylation (*hsdM*) subunits of the Sau1 type I restriction-modification system. A single copy of the gene for the restriction subunit is located elsewhere in the *S. aureus* chromosome [109]. The *hsdS* genes of the Sau1 system diverge significantly between members of different lineages and this determines variations in the sequences that will be specifically recognized as targets for modification through methylation. Since only modified sequences will be protected against restriction, exchange of DNA between members of same lineage will be allowed, while DNA transferred between isolates of different lineages will be digested. Because of this, the Sau1 system has been considered as a key factor in the control of lineage evolution.

**Figure 4:**
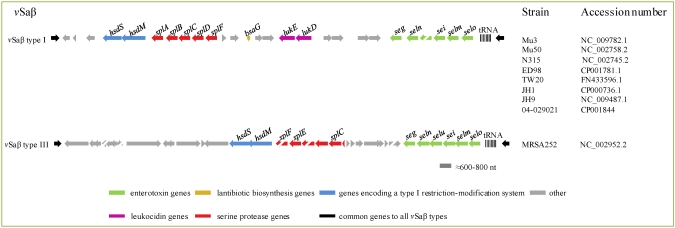
Structure of two types of the *v*Saβ genomic island containing the enterotoxin gene cluster. Adapted from Baba *et al.* [108] and based on accession numbers indicated to the right of the figure.

Both *v*Saα and *v*Saβ contain clusters of genes encoding known or putative virulence factors. *v*Saα carries a cluster of lipoprotein-encoding genes (*lpl* cluster), and the *set* (staphylococcal exotoxin-like) cluster [55,110], later re-named as the *ssl* (staphylococcal superantigen-like) cluster [30]. The *ssl* cluster consists of a series of related genes (between 7 and 11) coding for proteins that share a common architecture with SAgs but do not function as such [50]. However, they have alternative effects on the host immune system, acting on IgA, complement factor C5 (as demonstrated for SSL7; [53]), or neutrophils (SSL5 [111] and SSL11 [52]). *v*Saβ carries a serine protease gene (*spl*) cluster, genes for the components of the LukED leukocidin (*lukD* and *lukE*), genes for lantibiotic biosynthesis (*bsa*) and/or the enterotoxin gene cluster (*egc*), which includes a variable number of *se*/*sel* genes forming an operon [36]. Two representative types of vSaβ, the genomic island carrying *se* genes, are showed in Figure 4.

**Figure 5:**
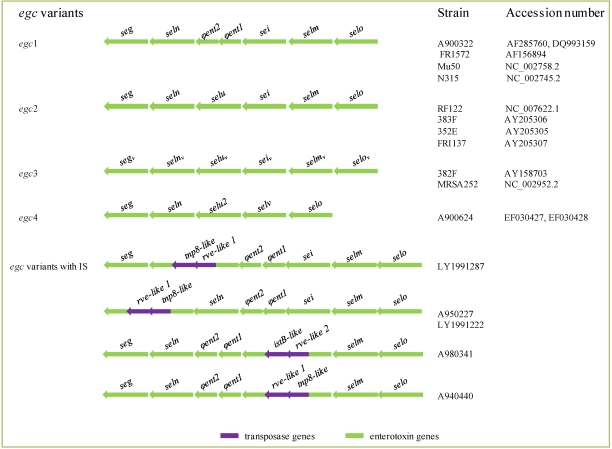
Structure of *egc* clusters. Modified from Thomas *et al* [28] and Collery *et al.* [114], and based on the accession numbers indicated to the right of the figure.

It has been suggested that the *egc* cluster arose from an ancestral *se* gene, through tandem duplication and further variation, while gene recombination has created variant toxins with different biological activities [28,36,112]. The dynamic evolution of this cluster that has been considered as a nursery of *se*/*sel* genes [36] is reflected in the number of variants already known (Figure 5).

The first *egc* (*egc1*) was discovered in 2001 and consists of two SE genes (*seg* and *sei*), three SE*l* genes (*selm*, *seln* and *selo*), and two pseudogenes (*φent1* and *φent2*) [36,113]. Afterward, a second *egc* variant (*egc2*) containing an additional SE*l* gene (*selu*) was described [37]. The latter gene has been generated by fusion of the two *egc1* pseudogenes, due to a 15 nucleotide insertion in *φent1* and a single adenine deletion that abolishes a stop codon within the same gene. In addition, allelic variants of each of the *egc2* genes compose the *egc3* cluster [37,114,115], and a new *selu* variant (*selu2*) and a novel *sel* gene (*selv*) are present in *egc4* [28]. A recombination event between *selm* and *sei* produced *selv*, while deletion of one adenine between the overlapping 5’ and 3’ ends of the *φent2* and *φent1* pseudogenes generated *selu2* (which was proposed to be renamed as *selw*) [116]. Incomplete *egc* clusters, lacking one or more genes of the classical *egc1*, as well as variants carrying insertion sequences within *seln*, *seg* or *sei*, have also been described [28,117]. These structures have been considered as evolutionary intermediates of the *egc* cluster [28]. Moreover, the fact that each of the three major homology groups of SEs/SEs (Table 2) contains enterotoxins encoded by genes of the *egc* operon led to the proposal that all *se*/*sels* originated from the *egc* cluster [29].

#### 2.4.5. Enterotoxin Genes in the Proximity of the Staphylococcal Cassette Chromosome

**Figure 6:**
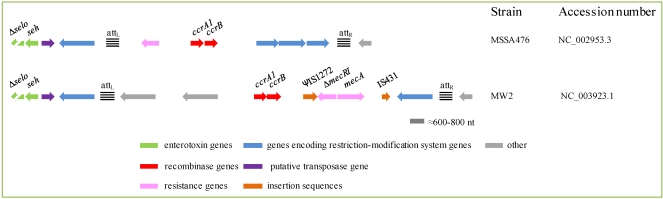
Comparison of two allelic forms of SCC elements associated with *seh*. Modified from Noto and Archer [118] and based on the accession numbers indicated to the right of the figure.

The *seh* gene, flanked by a truncated *selo* gene and a putative transposase gene, have been found in close proximity of the non-*mecA* containing SCC element harbored by MSSA (methicillin susceptible *S. aureus*) strain 476; the SCC*mec* type IV of *S. aureus* MW2; and the SCC*mec* type IV of a collection of highly related community-associated *S. aureus* ([118]; Figure 6). In the latter strains, acquisition of the *seh* element could have stabilized the integration of SCC*mec* type IV, which is unable to excise [118].

### 2.5. Staphylococcal Enterotoxins and Food Poisoning Outbreaks

Independently of their origin, enterotoxigenic *S. aureus* often differ in the number of mobile genetic elements and *se*/*sel* genes therein, as well as in the enterotoxins they produce. SEA, either alone or together with other SEs/SEls, is the enterotoxin most commonly reported in foods, and is also considered as the main cause of SFP, probably due to its extraordinarily high resistance to proteolytic enzymes [3,119,120]. The predominance of SEA is well documented in different countries. As relevant examples: (i) a comprehensive study of 359 outbreaks that occurred in the United Kingdom (UK) between 1969 and 1990 revealed that 79% of the *S. aureus* strains produced SEA [121]. Meat, poultry and their products, particularly ham and chicken, were the vehicle in 75% of the incidents. SEA was detected alone in 56.9% of the outbreaks and, in conjunction with SED, SEB, SEC or SEB and SED in a lower number of outbreaks (15.4, 3.4, 2.5 or 1.1%, respectively); (ii) SEA was also the enterotoxin most frequently found among 31 SFP outbreaks in France (69.7%), which were associated with a great variety of foods including milk products, different types of meat, and salads, between 1981 and 2002 [122]. In agreement with this, *sea* was the most common gene in the isolates tested, followed by *sed*, *seg*, *sei* and *she*; (iii) In Austria, an SFP outbreak that affected 40 children in 2007 was attributed to *S. aureus* isolates producing SEA and SED. Bovine milk products were identified as the source of the outbreak, and the cows, not the dairy owner, were the more likely reservoir of the SEs-producing *S. aureus* [123]; (iv) SEA was also the most common enterotoxin recovered from food poisoning outbreaks in USA (77.8% of all outbreaks) followed by SED and SEB [124]; (v) A study of *S. aureus* obtained from dairy products, responsible for 16 outbreaks in Brazil revealed that the most frequently encountered enterotoxin gene was *sea* followed by *seb* [125]. Finally, (vi) several studies have investigated the distribution of SEs and *se*/*sel* genes in *S. aureus* from foods and SFP outbreaks in Asian countries. Among strains recovered from patients associated with SFP outbreaks during 2001-2003 in Taiwan, *sea* was the most common gene, followed by *seb* and *sec* [13]. In Korea, about 90% of food poisoning isolates were reported to contain the *sea* gene [126]. SEA also was the most common SE associated to SFP in Japan [127]. In this country, an extensive outbreak that occurred in 2000 was attributed to low-fat milk containing SEA [128], while a recent outbreak (2009) was due to crepes containing SEA and SEC [129].

SEB, SEC or SED alone have been also implicated in SFP outbreaks through the world [121,122,125]. Interestingly, an outbreak, which affected three members of the same family in USA, was caused by coleslaw-containing SEC produced by a community-acquired methicillin resistant *S. aureus* from an asymptomatic food handler [130]. The fifth classical enterotoxin, SEE, has been infrequently reported in foods and food-producing animals, and its involvement in SFP outbreaks has only been demonstrated in rare occasions. However, six SFP outbreaks, which occurred in France at the end of 2009, were caused by SEE present in soft cheese made from unpasteurized milk. This enterotoxin has also been associated with outbreaks in USA and UK [33,121,131,132,133].

In contrast to classical SEs, the relationship between the novel SEs/SE*l*s and SFP is not fully understood. Among them, SEG, SEH and SEI, SER, SES, and SET have shown to be emetic after oral administration in a primate model, while the emetic activity of SE*l*L and SE*l*P has only been demonstrated in rabbits and the small insectivore *Suncus murinus*, respectively [39,43]. The remaining SE*l*s either lack emetic properties (SE*l*Q), or have not been tested (SE*l*J, SE*l*K, SE*l*M, SE*l*N, SE*l*O, SE*l*U, SE*l*U2 and SE*l*V). Moreover, commercial kits are not available for immunological detection of these SEs and SE*l*s, although ELISA (enzyme-linked immunosorbent assay) has been described for SEH [134] and for SEG and SEI [135]. Of the new enterotoxins, only SEH-producing strains have clearly been involved in SFP outbreaks [134,136,137,138], but results from different researchers have shown the high incidence of genes encoding new SEs and SE*l*s among food-borne *S. aureus* [131,139,140,141]. Mc Lauchlin *et al.* [131] revealed that 23 staphylococcal strains implicated in SFP outbreaks in UK, in which classical *se* genes were not detected, harbored one or more of the new *se*/*sel* genes, *i.e.*, *seg*, *seh*, *sei* or *selj*. It is possible that the corresponding SEs might have been the cause of these outbreaks. The presence of *egc* genes was also shown in food-associated *S. aureus* from other countries [131,140,141,142,143,144], and newly described SE or SE*l* genes, particularly those belonging to the *egc* cluster, were more frequently detected in *S. aureus* strains isolated from raw pork and chicken meat in Korea than genes encoding classical SEs [145]. Despite this, *egc*-encoded SEs or SE*l*s have not yet been directly implied in typical cases of SFP, although SEG and SEI have been reported as the cause of chronic diarrhea associated with severe but reversible enteropathy in two malnourished neonates [146].

## 3. Conclusions

SEs and SE*l*s produced by *S. aureus* belong to the fascinating family of superantigens, which sabotage the immune system of the host by targeting the innate and adaptive responses. Members of the family are well characterized with regard to superantigenic activity. However, the bases for the enterotoxigenic activity associated with a number of *S. aureus* superantigens remain elusive. Likewise, a direct relationship of *S. aureus* SEs (with demonstrated emetic activity) and SE*l*s (which lack emetic activity or have yet to be tested) with pathogenicity has not always been established, and the reasons for the redundancy of *se*/*sel* genes within the same bacterium deserve further attention. Of particular interest is the *egc* cluster, regarded as a nursery of *se*/*sel* genes in continuing evolution. The cluster and its multiple variants, located on the νSaβ genomic island, are widely distributed in *S. aureus* of any origin, and results from our group indicate that they are the most common superantigenes in *S. aureus* recovered from clinical samples, healthy carriers, cows with subclinical mastitis and foods [143,147,148,149]. However, a direct involvement of *egc-*encoded SEs in food poisoning has not been demonstrated, and attempts to elucidate their pathogenic role are still scarce [146,150,151,152]. In summary, although a wealth of information on SEs and SE*l*s is already available, they still represent an active field of research, which will certainly provide new exciting findings in forthcoming years.
